# Draft Genome Sequence of the Environmentally Isolated *Acinetobacter pittii* Strain IPK_TSA6.1

**DOI:** 10.1128/genomeA.01028-16

**Published:** 2016-09-29

**Authors:** Yunmi Lee, Soojin Jang

**Affiliations:** Discovery Biology Group, Antibacterial Resistance Research Laboratory, Institut Pasteur Korea, Gyeonggi-do, South Korea

## Abstract

*Acinetobacter pittii* is an opportunistic pathogen frequently isolated from *Acinetobacter* infections other than those from *Acinetobacter baumannii*. Multidrug resistance in *A. pittii*, including resistance to carbapenems, has been increasingly reported worldwide. Here, we report the 4.14-Mbp draft genome sequence of *A. pittii* IPK_TSA6.1 that was isolated from a nonhospital setting.

## GENOME ANNOUNCEMENT

*Acinetobacter pittii* is an aerobic Gram-negative bacillus that can be found in various settings, including soil, foods, sewage, and animals, as well as on human skin and flora (1–6). Although *A. pittii* is considered less virulent than *Acinetobacter baumannii* within the *Acinetobacter baumannii* complex, the significant role of *A. pittii* in human infections has been recognized (7–9). In particular, the emergence of carbapenem-resistant *A. pittii* strains possessing carbapenem-hydrolyzing β-lactamases, such as NDM1 and oxacillinases (OXA), has become a great medical concern (10–13).

The *A. pittii* strain used in this study was isolated from a microbiome collected from various surfaces of a building with <200 occupants in South Korea in 2015. Genomic DNA of the strain was extracted using the Wizard genomic DNA isolation kit (Promega, Madison, WI, USA) prior to preparation of a 20-kb library. By using the PacBio RS II (Pacific Biosciences, CA) sequencing platform, *A. pittii* IPK_TSA6.1 was sequenced and found to be 4,143,470 bp long in two contigs (4,027,663 bp and 115,807 bp, respectively). The overall G+C content was 38.96%. A total of 74,321 reads were assembled using the PacBio SMRT Analysis 2.3.0, providing 242.47-fold coverage of the genome. No plasmid was identified during the assembly and by PlasmidFinder, a Web-based *in silico* plasmid detection method (14). The gene annotation by the NCBI Prokaryotic Genome Annotation Pipeline (https://www.ncbi.nlm.nih.gov/genome/annotation_prok/) revealed 3,696 coding sequences with 95 RNA genes, including 73 tRNAs, four noncoding RNAs (ncRNAs), and six each of 5S rRNA, 16S rRNA, and 23S rRNA. Further investigation using an antibiotic resistance database (http://arpcard.mcmaster.ca) identified several potential antibiotic resistance genes, such as *ampC*, an ADC family cephalosporin-hydrolyzing class C beta-lactamase, and a gene encoding a homolog of beta-lactamase OXA-213 (15). The homolog of OXA-213 in *A. pittii* IPK_TSA6.1 has 99% nucleotide identification with and an identical amino acid sequence as OXA-502 in *A. pittii* strain NRZ_17616 isolated from Germany, except for two residues, S11 and E198, which are G11 and Q198 in NRZ_17616, respectively (accession numbers NG_049780.1 and ALP13526.1). The nearly identical sequences are interesting because they imply that the OXA genes in the two strains have the same origin and also suggest that *A. pittii* IPK_TSA6.1 has potentially intrinsic resistance to various beta-lactam antibiotics. The analysis of more genome sequences of *A. pittii* strains from various sources will help us better understand the species and possibly aid in the development of better therapeutic strategies for *A. pittii*-mediated infections.

### Accession number(s).

This whole-genome shotgun project has been deposited at DDBJ/ENA/GenBank under the accession no. LWHP00000000. The version described in this paper is version LWHP01000000.
